# Immunomodulatory effects of heat stress and lipopolysaccharide on the bursal transcriptome in two distinct chicken lines

**DOI:** 10.1186/s12864-018-5033-y

**Published:** 2018-08-30

**Authors:** Melissa S. Monson, Angelica G. Van Goor, Christopher M. Ashwell, Michael E. Persia, Max F. Rothschild, Carl J. Schmidt, Susan J. Lamont

**Affiliations:** 1 Department of Animal Science, Iowa State University, Ames, IA USA; 2 Department of Poultry Science, North Carolina State University, Raleigh, NC USA; 3 Department of Animal and Poultry Sciences, Virginia Polytechnic Institute and State University, Blacksburg, VA USA; 4 Department of Animal and Food Sciences, University of Delaware, Newark, DE USA

**Keywords:** Heat stress, Lipopolysaccharide, Chicken, RNA-seq, Transcriptome, Bursa

## Abstract

**Background:**

Exposure to heat stress suppresses poultry immune responses, which can increase susceptibility to infectious diseases and, thereby, intensify the negative effects of heat on poultry welfare and performance. Identifying genes and pathways that are affected by high temperatures, especially heat-induced changes in immune responses, could provide targets to improve disease resistance in chickens. This study utilized RNA-sequencing (RNA-seq) to investigate transcriptome responses in the bursa of Fabricius, a primary immune tissue, after exposure to acute heat stress and/or subcutaneous immune stimulation with lipopolysaccharide (LPS) in a 2 × 2 factorial design: Thermoneutral + Saline, Heat + Saline, Thermoneutral + LPS and Heat + LPS. All treatments were investigated in two chicken lines: a relatively heat- and disease-resistant Fayoumi line and a more susceptible broiler line.

**Results:**

Differential expression analysis determined that Heat + Saline had limited impact on gene expression (*N* = 1 or 63 genes) in broiler or Fayoumi bursa. However, Thermoneutral + LPS and Heat + LPS generated many expression changes in Fayoumi bursa (*N* = 368 and 804 genes). Thermoneutral + LPS was predicted to increase immune-related cell signaling and cell migration, while Heat + LPS would activate mortality-related functions and decrease expression in *WNT* signaling pathways. Further inter-treatment comparisons in the Fayoumi line revealed that heat stress prevented many of the expression changes caused by LPS. Although fewer significant expression changes were observed in the broiler bursa after exposure to Thermoneutral + LPS (*N* = 59 genes) or to Heat + LPS (*N* = 146 genes), both treatments were predicted to increase cell migration. Direct comparison between lines (broiler to Fayoumi) confirmed that each line had distinct responses to treatment.

**Conclusions:**

Transcriptome analysis identified genes and pathways involved in bursal responses to heat stress and LPS and elucidated that these effects were greatest in the combined treatment. The interaction between heat and LPS was line dependent, with suppressive expression changes primarily in the Fayoumi line. Potential target genes, especially those involved in cell migration and immune signaling, can inform future research on heat stress in poultry and could prove useful for improving disease resistance.

**Electronic supplementary material:**

The online version of this article (10.1186/s12864-018-5033-y) contains supplementary material, which is available to authorized users.

## Background

Negative impacts of heat stress on poultry health and performance have been well characterized and include decreased growth, feed efficiency, egg production, immune function, intestinal integrity, and even survival [1–4]. These effects will intensify as global climate change increases both the annual average temperature and the frequency of high temperature extremes [5]. Heat stress is estimated to cost the poultry industry millions of dollars each year [6]. This estimate would be even higher if the economic losses due to increased incidence of infectious diseases during heat stress could be included. One major form of immunosuppression in heat-stressed chickens is reduced humoral immunity, which can increase the risk of secondary infections and could also limit the efficacy of vaccination [1, 2, 4, 7].

The bursa of Fabricius is an avian-specific primary immune tissue connected to the cloaca that is responsible for B lymphocyte development and diversity in the antibody repertoire [8]. In chickens intensively selected for growth and muscle yield (broilers), chronic heat stress increases atrophy rate of the bursa and leads to atrophy of other immune tissues [2, 3, 7, 9]. Embryonic or post-hatch heat stress can decrease bursal follicle size and increase separation between follicles, which could impact B lymphocyte production [10–13]. Tang and Chen (2015) observed reductions in both blood and bursal B lymphocytes after cyclic heat stress [13]. Another study found that heat exposure prevented an increase in B lymphocyte numbers in the blood of broiler chickens in response to vaccination [14]. Although stimulatory effects on antibodies have been reported [14–16], elevated temperatures reduced circulating antibody levels in many other experiments [1, 2, 7, 9, 13]. Decreased antibodies have been observed after as few as four hours of acute heat stress [17].

As part of the gut-associated lymphoid tissue (GALT) [18], the bursa is likely to be impacted by heat-induced loss of intestinal integrity. In broilers, heat stress has been shown to increase intestinal permeability, inflammation, and infection with *Salmonella* [3, 4, 19–21]. Heat disrupts the tight junctions between enterocytes lining the intestinal tract and allows uptake of intestinal pathogens and molecules such as lipopolysaccharide (LPS) [22]. LPS is a gram-negative bacterial cell wall component that stimulates the immune system and, in large doses, can cause harmful systemic inflammation [22]. Similar to heat stress, experiments using LPS have shown adverse effects on the bursa, including tissue atrophy [23–25], and after embryonic exposure, reduced number of bursal follicles [26]. LPS is also well known to impact gene expression in chicken immune tissues, including at the transcriptome level [23, 27, 28].

Measuring global changes in gene expression can also identify genes and pathways involved in responses to or negatively affected by heat stress. Many experiments in chickens have investigated transcriptome responses to heat in diverse non-immune tissues [29–33]. However, the genes and pathways by which immune tissues respond to heat stress are not yet well studied on a genome-wide scale. Our group has previously examined heat stress and immune stimulation in chicken spleen using RNA-sequencing (RNA-seq) to measure transcriptome responses to acute heat and/or LPS [28]. Two distinct research lines of chicken maintained at Iowa State University (ISU) were included in the experiment. The inbred Fayoumi line was originally adapted to the high temperatures of Egypt [28, 34] and has been described as relatively resistant to pathogens such as *Salmonella*, Marek’s disease virus and Newcastle disease virus [35–37]. Generally more susceptible to heat and disease compared to the Fayoumi line, the broiler line was derived from a commercial outbred broiler breeder male line [28, 38].

In the current experiment, RNA-seq was used to characterize transcriptome responses to acute heat stress and/or subcutaneously administered LPS in the bursa of Fayoumi and broiler chickens. By incorporating these treatments in a 2 × 2 factorial design (*n* = 3–4 birds/treatment/line), our study can directly investigate the interaction of heat stress with an immune stimulus. We hypothesize that expression changes in the bursa will provide insight into the mechanisms by which high temperatures can affect bursacyte development, survival and migration. Understanding how heat stress changes bursal expression responses to LPS can provide informative genes and pathways to target for improvement of humoral-mediated disease resistance in the face of climate change.

## Results

### Dataset QC and mapping

Representing both research lines (broiler and Fayoumi) and all treatments (Thermoneutral + Saline, Thermoneutral + LPS, Heat + Saline, Heat + LPS), sequencing of 31 libraries (*n* = 3–4 samples/treatment/line) produced over 1.36 B single-end reads (Table 1, Additional file 1). Because raw read quality was already high, read trimming and filtering discarded only approximately 10% of each dataset and neither line (*p*-value = 0.24) nor treatment (*p*-value = 0.71) had a significant impact on the percentage of reads that passed QC. After QC, average sequencing depth was 39.5 M corrected reads/library (1.22 B total; Table 1, Additional file 1). Nearly 1.19 B corrected reads (96.9%) mapped to the chicken genome, of which 1.03 B (83.8% of corrected) mapped uniquely to exons of annotated genes (Additional files 1 and 2). Exonic mapping percentage was not impacted by line or treatment (*p*-values = 0.18 and 0.59, respectively). A small portion (1.8%) of reads aligned to multiple positions (not unique), while 11.3% aligned to intergenic regions (no feature) or to overlapping genes (ambiguous), providing evidence for unannotated genes and other novel expressed sequence in the chicken genome.

**Table 1:** Summary of bursal RNA-seq datasets

Read Status^a^	Mean Read Length (bp)	Mean Quality Score (Phred33)	Mean GC Content (%)	Number of Reads (M)	Total Sequence (Gb)
Raw	100.0	35.7	48.9	1363.9	136.4
Corrected	99.0	36.9	48.6	1223.8	121.2

^a^Before (raw) or after (corrected) read trimming and filtering

### Differential expression

**Fig. 1:**
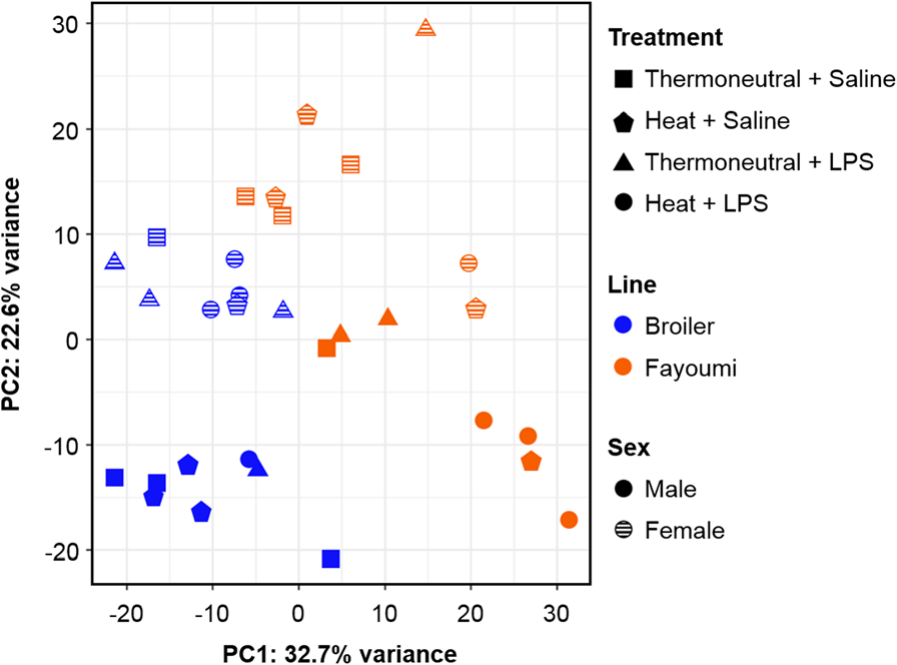
Bursal transcriptomes cluster by chicken line and sex. Principal component analysis (PCA) was performed on normalized variance stabilized read counts (from the 500 most variable genes) using the DESeq2 package [39]. The percent of variation explained by each component is shown in the axis titles. Datasets are distinguished by treatment (Thermoneutral + Saline (square), Heat + Saline (pentagon), Thermoneutral + LPS (triangle), Heat + LPS (circle)), line (broiler (blue), Fayoumi (orange)), and sex (male (solid), female (lined)). Lipopolysaccharide (LPS), principal component 1 (PC1), principal component 2 (PC2)

Considering only unique exonic mapped reads, 22,223 genes (91.1%) were expressed in the bursa, with more than 70% of the gene set observed in each bursal dataset (Additional file 1). Among the expressed genes, 16,780 had sufficient normalized read depth (counts per million > 0.25 in at least 3 datasets) for statistical analyses and were tested in DESeq2 [39]. Differential expression (DE) analysis incorporated effects of treatment, line, and sex, as principal component analysis (PCA) illustrated that both line and sex strongly contribute to transcriptome variation in the bursa (22–32%; Fig. 1). In total, 3580 genes had significant DE in at least one pairwise comparison (Additional file 3); 1833 genes had significant responses to treatment (within line), while 2747 were significantly different between lines (within treatment). Results of DE from 10 comparisons (between treatments) were validated using 16 genes in high-throughput quantitative PCR (qPCR); the correlation between log_2_ fold change (log_2_FC) obtained from DESeq2 and qPCR was 0.88 (R^2^ = 0.77; Additional file 4).

### Fayoumi responses to treatment

**Fig. 2:**
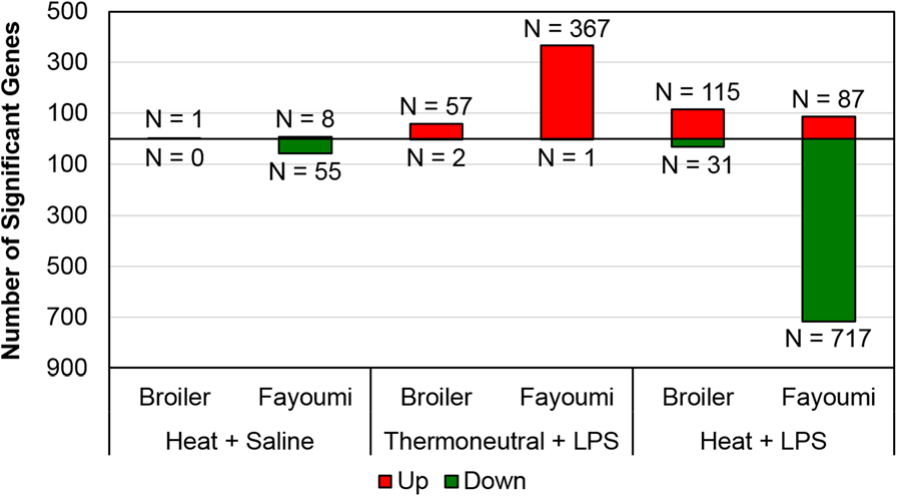
Significant changes in gene expression in response to heat stress and/or LPS. Genes with significant DE (q-value < 0.05, |log_2_FC| ≥ 1.0) were determined using DESeq2 [39] to perform pairwise comparisons (*n* = 3–4 samples/treatment/line) against Thermoneutral + Saline. Differential expression (DE), lipopolysaccharide (LPS), log_2_ fold change (log_2_FC), number of significant genes (N), up-regulated (red upward bars), down-regulated (green downward bars)

To characterize the response to heat stress and/or LPS within the Fayoumi line, expression in each treatment group was compared to the Thermoneutral + Saline control. A total of 1165 significant DE genes were identified in the Fayoumi bursa (Fig. 2, Additional file 3). Only 63 genes had significant expression changes in response to Heat + Saline, suggesting that baseline expression was largely maintained in the Fayoumi bursa during acute heat stress. Most responses (87.3%) to heat stress were down-regulatory, with the greatest decrease (negative log_2_FC) in cathelicidin-2 (*CATH2*) and ATP synthase membrane C locus 2 (*ATP5MC2*). Due to the small number of genes impacted by Heat + Saline in the Fayoumi bursa, no significant canonical pathway associations were made in IPA. After Thermoneutral + LPS treatment, 368 genes had significant DE and, conversely, all but one gene (uncharacterized *LOC112532754*) were increased by LPS. The largest responses (positive log_2_FC) were observed in somatostatin II (*SS2*), cysteine rich angiogenic inducer 61 (*CYR61*) and nuclear receptor subfamily 4 group A member 3 (*NR4A3*). Canonical pathway analysis in IPA predicted activation (*p*-value < 0.05; z-score ≥ 2) of multiple immune-related cell signaling pathways, including “Integrin Signaling”, “Signaling by Rho Family GTPases”, and “Leukocyte Extravasation Signaling” (Table 2).

**Table 2:** Predicted activation or inhibition of IPA canonical pathways in Fayoumi bursa

Canonical Pathways^a^	N^b^	Top Associated Genes^c^	z-score
Heat + Saline			
---	---	---	---
Thermoneutral + LPS			
GP6 Signaling Pathway	11	*COL2A1*, *LAMA2*, *CAMK4*, *COL6A3*, *COL4A6*	3.32
Integrin Signaling	11	*MYL9*, *ACTG2*, *ACTA2*, *MYLK*, *RND3*	3.00
Cardiac Hypertrophy Signaling	10	*CACNA1E*, *HSPB1*, *MYL9*, *PLCL1*, *CAMK4*	3.00
Calcium Signaling	11	*CACNA1E*, *CHRNA3*, *CHRNA7*, *TPM2*, *MYL9*	2.65
Signaling by Rho Family GTPases	15	*FOS*, *MYL9*, *ACTG2*, *DES*, *ACTA2*	2.50
Thrombin Signaling	10	*MYL9*, *PLCL1*, *MYLK*, *CAMK4*, *RND3*	2.45
Dopamine-DARPP32 Feedback in cAMP Signaling	8	*CACNA1E*, *PLCL1*, *KCNJ8*, *PRKG1*, *DRD1*	2.45
ILK Signaling	10	*FOS*, *MYL9*, *ACTG2*, *ACTA2*, *RND3*	2.33
Leukocyte Extravasation Signaling	8	*MMP1*, *ACTG2*, *CTNNA2*, *ACTA2*, *JAM3*	2.24
CXCR4 Signaling	7	*FOS*, *MYL9*, *EGR1*, *RND3*, *RHOB*	2.24
PI3K Signaling in B Lymphocytes	6	*FOS*, *ATF3*, *PLCL1*, *CAMK4*, *JUN*	2.24
Cholecystokinin/Gastrin-mediated Signaling	5	*FOS*, *RND3*, *RHOB*, *JUN*, *CREM*	2.24
Tec Kinase Signaling	6	*FOS*, *ACTG2*, *ACTA2*, *RND3*, *RHOB*	2.24
Intrinsic Prothrombin Activation Pathway	4	*COL2A1*, *COL1A2*, *COL1A1*, *COL3A1*	2.00
Agrin Interactions at Neuromuscular Junction	5	*LAMA2*, *ACTG2*, *ACTA2*, *AGRN*, *JUN*	2.00
Heat + LPS			
Superpathway of Cholesterol Biosynthesis	4	*PMVK*, *MVD*, *EBP*, *FDPS*	-2.00
Osteoarthritis Pathway	15	*BGLAP*, *GLI1*, *IL1B*, *HES1*, *SP1*	-2.11
Role of Wnt/GSK-3β Signaling in Pathogenesis of Influenza	6	*WNT6*, *WNT10A*, *WNT11*, *FZD2*, *WNT5B*	-2.24
Wnt/Ca+ pathway	6	*FZD2*, *WNT5B*, *PLCD4*, *PLCD3*, *RELA*	-2.45
PCP pathway	8	*WNT6*, *WNT10A*, *WNT11*, *EFNB1*, *JUND*	-2.82
Colorectal Cancer Metastasis Signaling	12	*WNT6*, *WNT10A*, *WNT11*, *PTGER2*, *IL6R*	-2.89
Glioblastoma Multiforme Signaling	9	*WNT6*, *WNT10A*, *WNT11*, *EGF*, *FZD2*	-3.00

^a^Pathways shown for each treatment have significant associations (−log(*p*-value) > 1.3) and predicted activation (z-score ≥ 2) or inhibition (z-score ≤ − 2)

^b^Total number of significant DE genes associated with each pathway (N)

^c^For each pathway, the five significant genes (q-value < 0.05) with the largest |log_2_FC| are shown

**Fig. 3:**
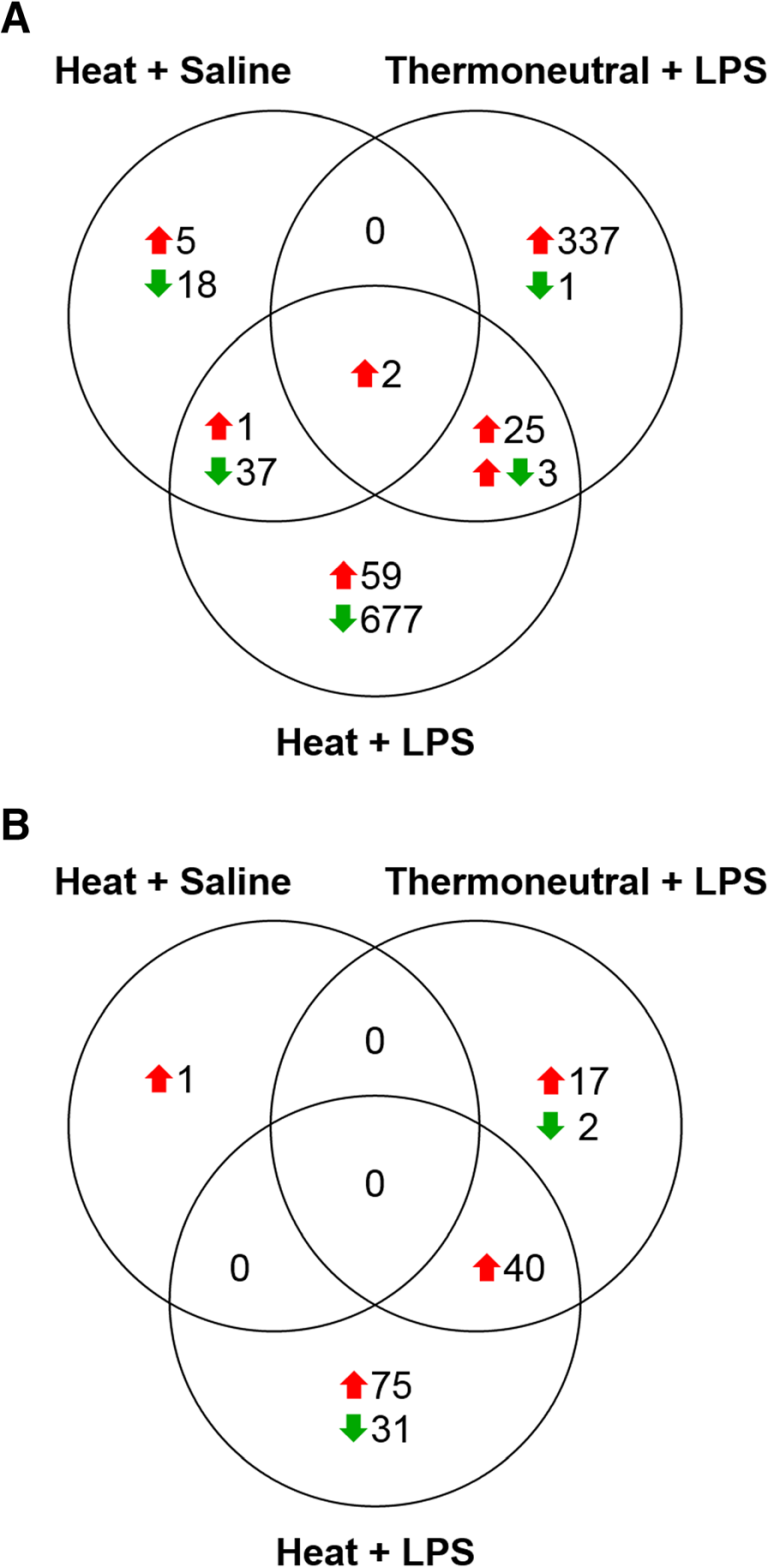
Minimal overlap in significant differential expression between treatments. **a**. Fayoumi. **b**. Broiler. Genes with significant DE (q-value < 0.05, |log_2_FC| ≥ 1.0) in pairwise comparisons to Thermoneutral + Saline were determined using DESeq2 [39]. Differential expression (DE), lipopolysaccharide (LPS), log_2_ fold change (log_2_FC), up-regulated (red upward arrow), down-regulated (green downward arrow), variable between contrasts (red upward arrow, green downward arrow)

The greatest number of significant DE genes (804) was detected in response to Heat + LPS; 89.2% of which had reduced expression in comparison to Thermoneutral + Saline (Fig. 2, Additional file 3). Dipeptidase 2 (*DPEP2*), potassium channel subfamily K member 4 (*KCNK4*), and cysteine-rich secretory protein LCCL domain containing 2 (*CRISPLD2*) were most up-regulated by Heat + LPS, while cysteine and histidine rich 1 (*CYHR1*), WNT family member 6 (*WNT6*), and WNT family member 10A (*WNT10A*) were most down-regulated. IPA predicted inhibition (*p*-value < 0.05, z-score ≤ − 2) of 7 canonical pathways, the top 5 of which include down-regulation of *WNT* genes (Table 2). There was limited overlap (8.2%) of significant DE from Thermoneutral + LPS with Heat + LPS, suggesting that heat stress prevented many responses to LPS in the Fayoumi bursa (Fig. 3a, Additional file 3). Although 27 of the shared genes were up-regulated by both treatments, three genes (protein tyrosine phosphatase receptor type Z1 (*PTPRZ1*), stathmin 3 (*STMN3*) and proteolipid protein 1 (*PLP1*)) had differing directions of DE, with up-regulation by Thermoneutral + LPS and down-regulation by Heat + LPS. Only *DPEP2* and *CRISPLD2* were significantly up-regulated in all three treatments.

**Fig. 4:**
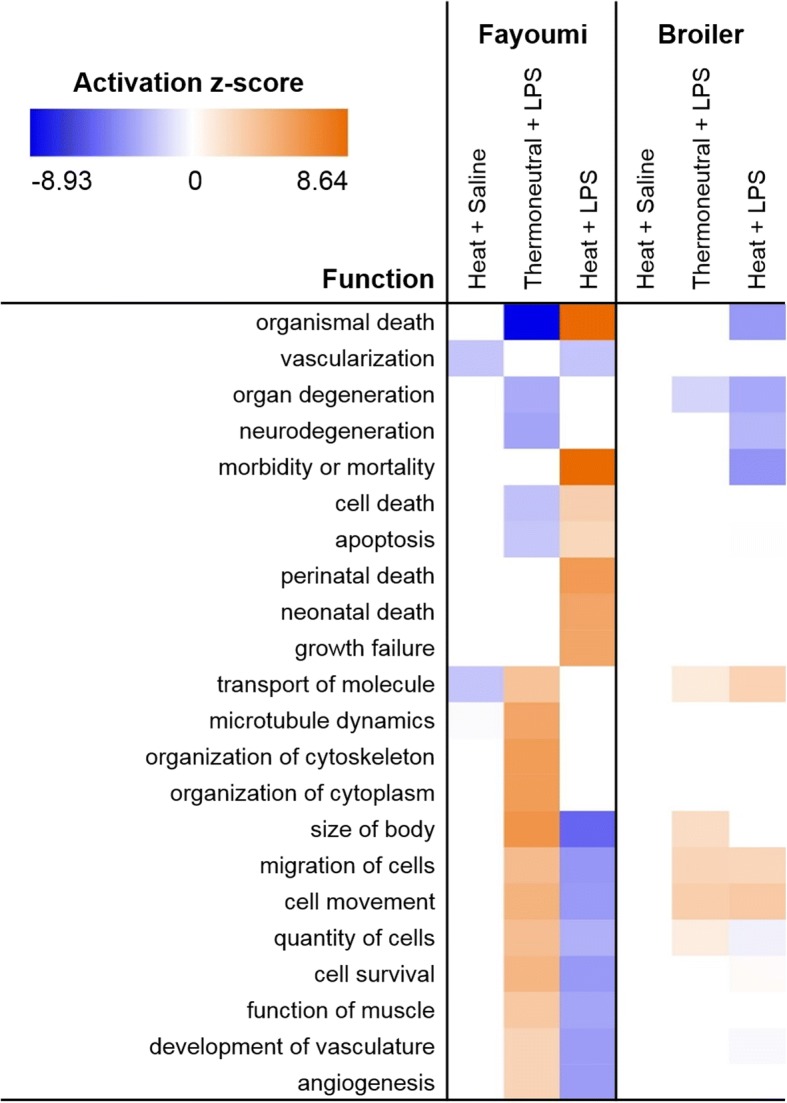
Predicted functional effects of differential expression in Fayoumi and broiler. Significant associations (−log(*p*-value) > 1.3) to potential gene functions were identified using Ingenuity Pathway Analysis (IPA) and z-scores were used to predict activation (z-score ≥ 2; orange) or inhibition (z-score ≤ − 2; blue) of each function. A subset of highly impacted functions are shown (|z-score| ≥ 5 or |z-scores| ≥ 2 in at least 2 comparisons). All activated or inhibited functions predicted for each comparison are given in Additional file 5. Lipopolysaccharide (LPS)

**Fig. 5:**
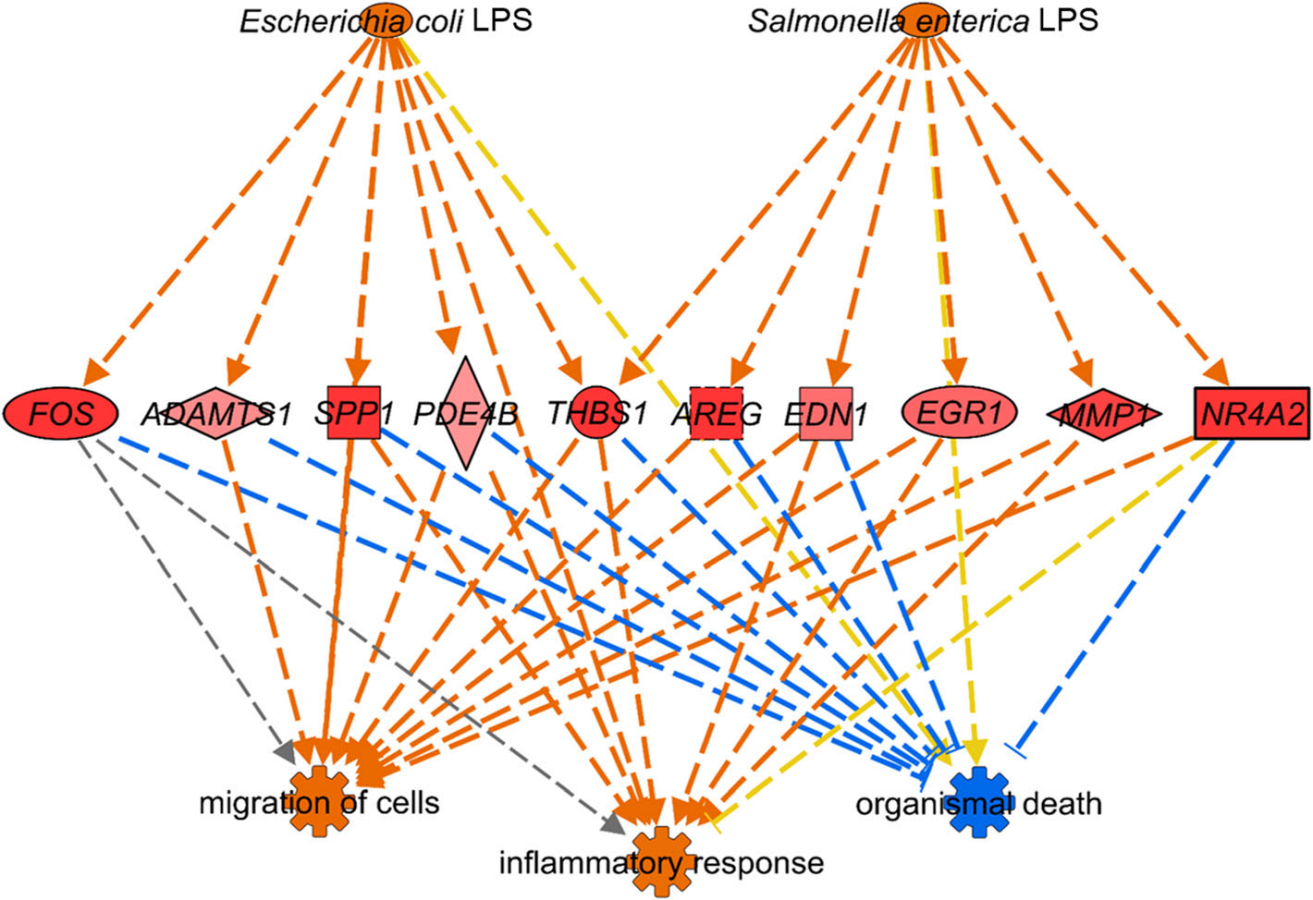
Up-regulation of LPS responsive genes in Fayoumi. IPA was used to identify a subset of genes known to respond to LPS with significant DE (q-value < 0.05, |log_2_FC| ≥ 1.0) in Fayoumi Thermoneutral + LPS. All ten genes were up-regulated by LPS (magnitude indicated by intensity of red color). Predicted relationships between these genes, LPS, and three down-stream functions include activation (z-score ≥ 2, orange), inhibition (z-score ≤ − 2, blue), not conclusive (gray), and inconsistent (yellow). Differential expression (DE), Ingenuity Pathway Analysis (IPA), lipopolysaccharide (LPS), log_2_ fold change (log_2_FC)

Prediction of the downstream functional effects of DE (z-scores) using IPA also demonstrated that there were opposing responses to Thermoneutral + LPS and Heat + LPS (Fig. 4, Additional file 5). Exposure to Thermoneutral + LPS caused significant DE in genes predicted to increase “size of body”, “organization of cytoskeleton”, “organization of cytoplasm”, and “microtubule dynamics”, while DE was predicted to decrease “organismal death”. Additionally, multiple genes known to respond to LPS in other species were significantly up-regulated by Thermoneutral + LPS (including nuclear receptor subfamily 4 group A member 2 (*NR4A2*) and thrombospondin 1 (*THBS1*)) and were linked to increased “inflammatory response” and “migration of cells” using IPA (Fig. 5). None of these genes had significant DE when the birds were exposed to heat stress before LPS. Opposite of Thermoneutral + LPS, Heat + LPS led to strong predictions of increased “organismal death” and “morbidity and mortality” (Fig. 4, Additional file 5).

### Broiler responses to treatment

A much smaller number of genes (166) had significant DE in the broiler bursa than the Fayoumi bursa after heat and/or LPS treatment (Fig. 2, Additional file 3). USH1 protein network component harmonin (*USH1C*) was the only gene with significant DE (increased) after Heat + Saline exposure; this suggests that basal expression levels in broiler bursa were nearly unaffected by the acute heat stress. There were 59 significant genes in response to Thermoneutral + LPS, with largest up-regulation in *KCNK4* and pyruvate dehydrogenase kinase 4 (*PDK4*). Only a major histocompatibility complex class I F10 alpha chain-like gene (*LOC107054704*) and chromosome 1 open reading frame human CXORF36 (*C1HXORF36*) had decreased expression in response to Thermoneutral + LPS. Heat + LPS again generated the greatest number of significant genes (146) and, unlike in Fayoumi, most (78.8%) of these genes had increased expression. The largest up-regulation was observed in cochlin (*COCH*) and heat shock family B member 9 (*HSPB9*), while arylsulfatase family member I (*ARSI*) and a feather keratin 1-like gene (*LOC769139*) were most down-regulated.

In broiler, approximately 2/3 of the significant genes in Thermoneutral + LPS were also observed in Heat + LPS (Fig. 3b). Eight of these genes (*KCNK4*, nuclear factor interleukin 3 regulated (*NFIL3*), FK506 binding protein 5 (*FKBP5*), transmembrane protein 88B (*TMEM88B*), LHFPL tetraspan subfamily member 5 (*LHFPL5*), *DPEP2*, carboxymethylenebutenolidase homolog (*CMBL*) and an uncharacterized ncRNA (*LOC107054792*)) were also significantly up-regulated in Fayoumi in both Thermoneutral + LPS and Heat + LPS, illustrating a few conserved responses to LPS regardless of heat treatment (Additional file 3). The single significant gene in broiler Heat + Saline, *USH1C*, did not respond to any other treatment. Unlike in Fayoumi, IPA down-stream functional predictions in broiler showed activation of “cell movement” and “migration of cells” in both Thermoneutral + LPS and Heat + LPS and an inhibition of “morbidity or mortality” in Heat + LPS (Fig. 4, Additional file 5). However, due to the small numbers of DE genes in broiler, IPA did not predict activation or inhibition of any canonical pathways in any treatment.

### Inter-treatment comparisons

The effects of heat stress on responses to LPS were further investigated through direct inter-treatment comparisons (Heat + LPS compared to Heat + Saline or Heat + LPS compared to Thermoneutral + LPS). In Fayoumi, 34 genes had significant expression changes in the comparison of Heat + LPS to Heat + Saline (Additional file 3). A far greater 1251 genes had significant DE in Heat + LPS compared to Thermoneutral + LPS; only 9 of these genes were up-regulated, illustrating that adding heat stress to LPS stimulation had a down-regulatory effect in the Fayoumi bursa. Of these genes, 523 (41.8%) were unique to the inter-treatment comparison (not significant in Thermoneutral + LPS or Heat + LPS compared to Thermoneutral + Saline); these genes represent divergence of Heat + LPS and Thermoneutral + LPS with intermediate expression levels in Thermoneutral + Saline.

Further interpretation of the combination of heat stress and LPS (Heat + LPS compared to Thermoneutral + LPS) can be made through the non-unique genes significant in the comparisons to Thermoneutral + Saline. Heat + LPS can suppress, not affect, or induce DE in these genes; see examples of genes in each of these categories in Additional file 6. Nearly 200 genes shared between the inter-treatment comparison and Thermoneutral + LPS compared to Thermoneutral + Saline were suppressed by Heat + LPS (Table 3, Additional file 3). These genes were up-regulated in response to Thermoneutral + LPS, but Heat + LPS reduced expression to basal levels (196 genes) or led to significant down-regulation (3 genes). Therefore, many up-regulatory responses to LPS did not occur if birds were already exposed to heat stress. The remaining 529 significant genes were shared by the inter-treatment comparison and Heat + LPS compared to Thermoneutral + Saline, illustrating that Heat + LPS induced DE in genes that did not respond to heat stress or LPS alone. Excluding the uncharacterized ncRNA *LOC101749104*, these genes were down-regulated in both Heat + LPS and the inter-treatment comparison.

**Table 3:** Interactions between heat stress and LPS differed between lines

Type of DE Interaction^a^	Significant			Number of Genes			
	Inter-treatment^b^	Single treatment^c^	Heat + LPS^d^	Heat + LPS to Heat + Saline		Heat + LPS to Thermoneutral + LPS	
				Broiler	Fayoumi	Broiler	Fayoumi
Suppressed	Y	Y	N/Y	1	0	0	199
Unaffected	N	Y	Y	0	40	40	27
Induced	Y	N	Y	95	22	1	529

^a^Inter-treatment comparisons (Heat + LPS to Heat + Saline or to Thermoneutral + LPS) identified three effects of combined treatment on DE (suppressed, unaffected, or induced). See Additional file 6 for examples of each interaction type

^b^Heat + LPS to Heat + Saline or Heat + LPS to Thermoneutral + LPS

^c^Heat + Saline to Thermoneutral + Saline or Thermoneutral + LPS to Thermoneutral + Saline

^d^Heat + LPS to Thermoneutral + Saline

Differential expression (DE), lipopolysaccharide (LPS), yes (Y), no (N)

In broiler, inter-treatment comparisons identified 149 significant genes in Heat + LPS to Heat + Saline and only one gene in Heat + LPS to Thermoneutral + LPS (Additional file 3). These expression patterns demonstrate that there is more similarity in broiler responses to Heat + LPS and to Thermoneutral + LPS, than to Heat + Saline. Again representing divergence of the treatments, 53 genes were unique to the inter-treatment comparison of Heat + LPS to Heat + Saline. Among the non-unique genes, the greatest number (95) was induced by Heat + LPS (significant in Heat + LPS compared to Heat + Saline and in Heat + LPS compared to Thermoneutral + Saline), and showed the same direction and similar magnitude of expression in both comparisons (Table 3, Additional file 3). The only significant gene in Heat + Saline to Thermoneutral + Saline, *USH1C*, was down-regulated in Heat + LPS compared to Heat + Saline, suggesting that this change was suppressed by LPS.

### Comparison between lines

To examine the distinct responses in each chicken line, direct comparisons of the broiler to Fayoumi were made within each treatment. In the control (Thermoneutral + Saline), there were 848 significant DE genes due to baseline differences in bursal expression between lines (Additional files 3 and 7). Functional predictions using IPA indicated that broiler may have reduced “transport of molecule” and “synaptic transmission of nervous tissue” compared to Fayoumi, which suggested that the constitutive expression levels of genes that can impact responses to stressors like heat and LPS may be different between these research lines. Considering only genes that did not have these homeostatic differences, there were 472, 1125, and 621 genes expressed significantly differently between lines in Heat + Saline, Thermoneutral + LPS, and Heat + LPS (Additional files 3 and 7).

These subsets of genes reflected variation in how the lines respond to heat stress or LPS. In response to Heat + Saline or Thermoneutral + LPS, IPA functional prediction suggests that there would be more cell movement in Fayoumi than broiler, while conversely, Heat + LPS could lead to higher cell movement in broiler than Fayoumi (Table 4). Heat + LPS is also predicted to cause neoplasia and cell degeneration in Fayoumi. These patterns are consistent with the results of the pairwise treatment comparisons (within line). The associations to cell movement in Heat + LPS were driven by 66 genes with significant DE between lines; 83.3% of these genes had increased expression in the broiler compared to Fayoumi, including 12 genes encoding regulators of transcription. Also included in this set of genes, C-reactive protein (*CRP*) and interleukin 6 receptor (*IL6R*) were increased in broiler compared to Fayoumi under Heat + LPS, while multiple MHC class I-like genes were decreased (Additional file 3).

**Table 4:** Predicted functional effects of treatment were different in broiler and Fayoumi lines

Treatment^a^	Fayoumi > Broiler	Broiler > Fayoumi
Heat + Saline	• cell movement	• movement disorders • mass of organism • colitis • abnormality of large intestine • skin lesion
Thermoneutral + LPS	• size of body • organization of cytoskeleton • organization of cytoplasm • microtubule dynamics • cell movement	• organismal death • morbidity and mortality • perinatal death • neonatal death • bleeding
Heat + LPS	• neoplasia of leukocytes • neurodegeneration	• cell movement • invasion of carcinoma cell lines • cell movement of carcinoma cell lines • migration of carcinoma cell lines

^a^Down-stream effects of each treatment were predicted using IPA (−log(*p*-value) > 1.3, |z-scores| ≥ 2). Top five functions (greatest |z-scores|) are shown for each line comparison. Analyses excluded basal line differences (848 genes significant in the comparison between lines for Thermoneutral + Saline; Additional files 3 and 7)

## Discussion

As a primary immune tissue, the bursa is responsible for the development of pre-B cells, repertoire diversity through the processes of V(D)J recombination and gene conversion, and ultimately export of naïve B cells to the periphery [8]. Effects on the bursa under heat stress could be due to reduced intestinal integrity increasing exposure to pathogens and antigenic molecules, such as LPS, or systemic stress responses such as circulating cytokines and acute phase proteins. Together these could impact bursacyte development, survivability and migration. Approximately 60% of circulating B lymphocytes in 21 day old chickens are recent emigrants from the bursa that can survive for a maximum of 3 days [8, 40, 41]. Therefore, at the time point investigated in this experiment, sufficient B cell export from the bursa is critical to maintaining a normal peripheral B cell population and potential humoral immune responses.

Identifying genes and pathways in primary immune tissues and the periphery that are involved in immunomodulation during heat stress can provide targets for understanding and improving disease resistance in heat-stressed birds. To this end, this study used transcriptome analysis to measure bursal responses to an acute heat stress and/or LPS and the resulting expression patterns illustrated that responses in the bursa were dependent on both chicken line and treatment. Gene expression in spleen and heterophils and phagocytic ability and other immune functions of heterophils have demonstrated that immune responses differ between the Fayoumi and broiler lines [28, 36, 42–45]. Direct comparison of the bursa from these lines has not been previously performed; this bursal transcriptome analysis identified line differences in baseline expression and responses to treatment. A larger number of significantly differentially expressed genes was identified in the Fayoumi line than the broiler line, perhaps reflecting the difference in variability between these research lines. As the Fayoumi line was highly inbred (> 99%) while the broiler line was derived from an outbred population [34, 38], the higher variation in the broiler line could decrease the ability to detect differential expression.

*CRISPLD2* was one of only two genes significantly up-regulated in the Fayoumi line across all three treatments; mammalian CRISPLD2 contains two LCCL domains that allow it to bind to LPS and prevent activation of the immune system [46]. Anti-inflammatory roles for CRISPLD2 have been further suggested by work in the human lung [47, 48]. Up-regulation of *CRISPLD2* in the chicken bursa by either heat or LPS is the first evidence suggesting that CRISPLD2 plays a similar role in the chicken GALT. Unlike *CRISPLD2*, most bursal expression responses were variable between treatments. Compared to the treatments involving LPS, exposure to 7 h of heat stress had minimal effects on bursal expression in either chicken line, suggesting that this tissue was able to maintain baseline expression levels and, thus, may be fairly robust to acute heat. This is in agreement with previous phenotypic findings, where lesions in the bursa required longer or repeated exposures to high temperatures [10, 12, 13]. In Fayoumi bursa, the largest impact of acute heat stress was decreased expression of *CATH2*. Cathelicidin genes encode host defense proteins that have antimicrobial activity [49–53] and can bind LPS as a means to reduce the inflammatory response [52–54]. Decreased expression of this gene, if translated to the protein level, could impact the innate immune response and increase inflammation. Based on analysis of another tissue from the same individuals, acute heat stress had more impact on expression in the spleen than seen here in the bursa [28].

Bursal responses to a large dose of LPS (50 mg/kg) have previously been characterized by RNA-seq in broilers and identified only 22 of the genes that responded in the current study using a lower dose of LPS (100 μg/kg) [23]. This includes the innate immune genes S100 calcium binding protein A9 (*S100A9*) and complement component 7 (*C7*), as well as two of the most up-regulated genes in the Fayoumi Thermoneutral + LPS response, *CYR61* and *NR4A3*. In the current bursal transcriptome analysis, exposure to LPS was associated with predicted increases in cell migration and cytoskeletal remodeling, as well as predicted activation of the “Leukocyte Extravasation Signaling” pathway in the Fayoumi bursa. The “Granulocyte Adhesion and Diapedesis” pathway was associated with the response to LPS exposure in the Fayoumi spleen [28]. Cell migration predictions in the bursa could be indicative of LPS increasing B lymphocyte trafficking to the periphery or could reflect an influx of cells into the bursa. The first hypothesis is more likely, as subcutaneous LPS was a systemic immune stimulus, not local to the bursa. Bursal exposure to LPS is also possible as a result of heat stress disruption of the intestinal barrier; this exposure would be a component of the Heat + Saline response, which had limited impact on expression.

LPS responses in Fayoumi also increased expression of three related genes, nuclear receptor subfamily 4 group A member 1 (*NR4A1*), *NR4A2* and *NR4A3*; these orphan nuclear receptors are transcription factors, but their endogenous ligands remain unknown [55]. All three *NR4A* genes are known to be up-regulated by LPS in human and murine myeloid cells [55–58]. Mammalian NR4A proteins act as regulators of the NF-κB pathway, with predominantly anti-inflammatory effects [55–58]. These proteins are also involved in neutrophil survival [59], regulatory T lymphocyte development [60, 61], and cell migration [55, 62, 63]. The chicken *NR4A* family has begun to be investigated in neurons and skeletal muscle, but immune-related functions in birds remain to be elucidated [64, 65]. Up-regulation of the *NR4A* genes in the chicken bursa could be indicative of increased lymphocyte trafficking after LPS exposure, although the *NR4A* genes may negatively regulate other inflammatory processes.

Compared to each single treatment, a larger number of expression changes was identified in response to Heat + LPS in both broiler and Fayoumi bursa. In the analysis of spleens from these same individuals, Heat + LPS also led to the greatest response in both lines [28]. Approximately 10% of the significant DE genes in bursa in response to Heat + LPS were also significant in the spleen after Heat + LPS, suggesting that there are stress responses that may be conserved across tissues. Interestingly, in the spleen after Heat + LPS exposure, heat shock 70 kDa protein 2 (*HSPA2*) was extremely down-regulated in both lines, while this gene was up-regulated by Heat + LPS in the Fayoumi bursa. *HSPA2* encodes an important heat shock protein (hsp70) that is often utilized as an indicator of stress and has been shown to have increased mRNA expression in the bursa in response to heat stress [66, 67] and in a B cell line after LPS stimulation [68]. In vitro experiments also observed up-regulation of *HSPA2* in response to a combined heat and LPS treatment in Fayoumi BMDCs [69].

Most expression changes in the Fayoumi bursa in response to Heat + LPS were down-regulatory. Pathway analysis predicted that this would have an inhibitory effect on WNT signaling, with reduced expression of *WNT6*, *WNT10A*, WNT family member 11 (*WNT11*), and WNT family member 5B (*WNT5B*). Only *WNT10A* decreased significantly in broiler. Human orthologues for two of these genes (*WNT5B* and *WNT10A*) are known to be expressed in B lymphocyte progenitors in bone marrow [70, 71]. *WNT* genes encode extracellular proteins that bind to frizzled receptors and activate pathways that regulate lymphocyte development [70]. In mammals, the WNT-Ca^2+^ pathway has been shown to inhibit developing B lymphocytes; *WNT5B*, *WNT6*, and *WNT11* signal through this pathway and may have similar suppressive effects [70, 72]. Conversely, mammalian *WNT10A* signals through the canonical WNT pathway, which generates proliferative signals for B lymphocytes [70, 73]. Therefore, in the Fayoumi bursa, predicted decreases in WNT signaling in response to Heat + LPS could either decrease B cell proliferation, or inversely, reduce an inhibitory mechanism to try to increase B cell survival despite the stress.

Nearly 200 genes that responded to Thermoneutral + LPS were suppressed by Heat + LPS in Fayoumi, illustrating that pre-treatment with heat stress may have prevented LPS responses in the Fayoumi bursa. Although less responsive to any treatment, the broiler did not show this negative interaction between heat and immune stimulation, with predictions of cell movement in response to either Thermoneutral + LPS or Heat + LPS. This is contrary to expectation, as the Fayoumi line is known to be more resistant to multiple diseases and to be more heat tolerant [28, 30, 35–37]. However, other hypotheses could explain the predicted differences in broiler and Fayoumi. Baseline differences between the research lines at the protein or metabolite level could allow the Fayoumi line to be more prepared to respond to these stressors. Thus, after Heat + LPS, only the broilers needed to induce gene expression in cell movement pathways. It is also possible that less inflammation and cellular damage developed in the Fayoumi line so there was less signal to induce gene expression. Lastly, these expression changes might be modulating the regulation of these processes in Fayoumi, leading to reduced expression, rather than actually reducing cell trafficking.

Further research is still necessary to reach the goal of increased disease resistance in heat-stressed poultry. Characterization of the effects of heat stress on lymphocyte survival and proliferation in different immune contexts, including ex vivo, and a combination of histology and functional assays would be needed to determine if there are negative impacts on bursacytes, but not mature B lymphocytes in the spleen or periphery. For example, determining the functional impacts of *WNT* knockdown or *NR4A* overexpression on B cells could verify the importance of these pathways and whether they have a positive or negative effect on immune capabilities. To extend the gene expression patterns observed in this study, there is need to examine multiple time points and to investigate commercial poultry populations. The predicted decreases in cell migration in the Fayoumi line could also be reflective of a mechanism to mitigate over-inflammation; cell movement and inflammatory responses were not so modulated in the broiler. Connected with this, the down-regulatory effects of Heat + LPS in the Fayoumi line compared to the broiler line could also be related to differences in the homeostatic expression of immune genes and stress response proteins in each line. If future investigation of basal gene or protein expression in these pathways identified higher levels in Fayoumi, there would be no need to increase expression, even during stress. Further investigation of expression and functional changes in cell proliferation and migration will be needed to fully characterize the interaction of heat stress with immune stimulation.

## Conclusions

Transcriptome analysis characterized genes and pathways that respond to heat stress and/or LPS in a primary immune tissue unique to birds. Acute heat stress induced only a few significant expression changes in the bursa of broiler and Fayoumi chickens. Exposure to LPS had a larger impact on both lines and was predicted to increase cell migration, perhaps modulating B lymphocyte trafficking. In Fayoumi bursa, addition of heat to LPS suppressed expression of many LPS responsive genes, although the down-regulation of WNT signaling genes in this combined treatment could positively affect bursal functions. Significant genes identified in this analysis, such as the *WNT* and *NR4A* genes, can provide targets for further research into resistance to heat and infectious disease; potential avenues of investigation include the effects of gene modulation on B cell functions and humoral immunity, confirmation of responses in commercial poultry populations, and identification of segregating variation in the relevant populations. Genes associated with cell movement and immune signaling could be promising candidates for selection to improve humoral-mediated immune responses in heat-stressed poultry.

## Methods

### Animals

The Institutional Animal Care and Use Committee at Iowa State University (ISU) approved all animal experiments (protocol # 4-11-7128-G). Chicks from a closed broiler line and an inbred Fayoumi line maintained at ISU were utilized in this study [28, 34, 38]. Birds were raised in floor pens with ad libitum access to feed (corn-soy based) and water throughout the entire experiment. Birds were vaccinated for Marek’s disease virus on day of hatch according to the manufacturer’s standard protocol (MD-Vac CFL, Zoetis Inc., Florham Park, NJ, USA).

### Acute heat stress and immune stimulation

Two independent replicates (C and E) of this experiment were performed, in which birds from each line (*n* = 26 broilers, 23 Fayoumi) were assigned to four treatments: Thermoneutral + Saline, Thermoneutral + LPS, Heat + Saline, and Heat + LPS. In each replicate, birds were transferred at 17 days of age into four temperature controlled chambers and placed into 2–4 pens in each chamber. All birds were allowed to acclimate to the pens for 5 days at a temperature of 25 °C. On day 22 of age (including acclimation period), the temperature in two chambers was increased to 35 °C to provide an acute heat stress (ramping from 25 °C to 35 °C took 30–40 min). The other chambers remained at 25 °C throughout the experiment. At 3.5 h after the start of heat stress, birds in the Heat + LPS (*n* = 16) and Thermoneutral + LPS (*n* = 8) groups were stimulated with a subcutaneous injection of lipopolysaccharide (LPS) derived from *Salmonella enterica* serotype typhimurium (L7261, Sigma-Aldrich, St. Louis, MO, USA). LPS stimulation was based on Kaiser et al., 2012, which detected differential cytokine expression in broiler spleen 3 h after exposure to 100 μg/kg LPS [74]. In the current study to account for differences in body size between lines, a dose of 100 μg of LPS/kg of body weight (using average weight for each line at day 21) was given in a total volume of 100 μL. Concurrently, birds in the Heat + Saline (*n* = 16) and Thermoneutral + Saline (*n* = 9) groups were injected with an equivalent volume (100 μL) of phosphate buffered saline. Each pen contained birds receiving saline and birds receiving LPS to minimize housing effects. After an additional 3.5 h (7 h into heat stress), birds were euthanized by intravenous injection of sodium pentobarbital (Sleepaway, Fort Dodge Animal Health, Fort Dodge, IA, USA). Tissue samples from the bursa were collected into RNAlater (Ambion, Inc., Austin, TX, USA), perfused for 24 h at room temperature, and then stored at − 80 °C to preserve the RNA.

### RNA isolation and sequencing

The bursa tissues used in the current study were obtained from the same individuals that provided spleens for Van Goor et al., 2017 [28]. Tissues (*n* = 4 samples/treatment/line; total *n* = 32) were homogenized using a Polytron homogenizer (Brinkmann, Mississauga, ON, CAN) and RNA isolated using the RNAqueous Total RNA Isolation kit (Ambion, Inc.) according to the manufacturer’s standard protocol. All RNA samples were DNase treated using the DNA-*free*™ DNA Removal kit (Ambion, Inc.). RNA quality and concentration were initially measured by spectrophotometry on the Nanodrop 1000 (Nanodrop Technologies, Wilmington, DE, USA) and then verified using the Eukaryote Total RNA Nano chip on the 2100 Bioanalyzer (Agilent Technologies, Santa Clara, CA, USA). RNA samples (*n* = 31) selected for library construction were high quality (RNA Integrity Number (RIN) ≥ 8.9; average RIN = 9.6); all 4 samples/treatment/line were utilized, with the exception of the Fayoumi Thermoneutral + LPS group (*n* = 3), where sufficient high quality RNA could not be generated for one tissue sample.

Individual barcoded cDNA libraries were generated from 0.5 μg of total RNA/sample using the Illumina TruSeq RNA Library Preparation kit version 2 (Illumina, Inc., San Diego, CA, USA). See Additional file 1 for experimental and bioinformatic information for each individual library. Quality of cDNA libraries was confirmed on the 2100 Bioanalyzer (Agilent Technologies) using the DNA 1000 chip. Libraries were submitted to the ISU DNA Facility for sequencing on the HiSeq 2500 (Illumina, Inc.). The flowcell was run in high output mode and produced 100 bp single-end reads. Each combination of treatment and line was represented within every lane (*n* = 8 samples/lane, see Additional file 1 for lane assignments) and all libraries were sequenced in two separate lanes to provide technical replicates (*n* = 8 lanes).

### Dataset QC and read mapping

RNA-seq datasets were de-multiplexed (see Additional file 1 for individual barcodes) and raw reads were filtered and trimmed using Trimmomatic version 0.32 [75] and the FastX Toolkit [76]. Within Trimmomatic, Illumina TruSeq adapters were removed using Illuminaclip (maximum seed mismatch = 2, simple clip threshold = 10), low quality bases were trimmed using Slidingwindow (window size = 4, minimum average quality score = 15), and reads less than 40 bp in length were discarded (Minlen). Datasets were then passed through the FastX Quality Filter tool (minimum quality score = 30, minimum percentage of bases with this quality = 80) to ensure overall read quality. FastQC version 0.10.1 was used to compare dataset quality before and after read processing [77]. Corrected reads were mapped onto the chicken reference genome (Galgal6a, GCA_000002315.5, NCBI Annotation Release 104; 24,403 genes) using STAR version 2.5.3a (genome indices generated by runMode = genomeGenerate, alignment performed with runMode = alignReads, outSAMtype = BAM SortedByCoordinate) [78, 79]. Mapped reads were sorted by name using samtools version 1.8 [80] and then counted by gene using HTSeq version 0.9.1 (feature type = exon, feature attribute = gene_id, mode = intersection nonempty) [81].

### Differential expression analysis

Significant differential expression (DE) of genes in pairwise comparisons between groups (group = line + treatment) was determined using DESeq2 version 1.20.0 [39]. Genes were filtered for sufficient normalized read depth (counts per million > 0.25 in at least 3 datasets) prior to statistical analysis. Following the standard workflow, read counts in each library were normalized for differences in sequencing depth across all libraries, used to estimate dispersions from the mean, and fit to a generalized linear model based on a negative binomial distribution. To identify the factors to incorporate into the model, principal component analysis (PCA) was performed using a variance-stabilized transformation of the normalized read counts (from the 500 most variable genes). The selected model incorporated the main effect of group and accounted for secondary effects of sex (design = ~ sex + group). Log_2_FC for each gene in each comparison were calculated using normalized read counts and a zero-centered prior; this shrinkage method minimizes over-dispersion and increases the reproducibility of log_2_FC values. Wald inferences tests were used to assign False Discovery Rate (FDR)-adjusted *p*-values (q-values) for each gene in each pairwise contrast. Both significance level (q-value < 0.05) and magnitude of expression change (|log_2_FC| ≥ 1.0) were used to select significant DE genes.

### Pathway analysis

Functional analyses were performed using Ingenuity Pathway Analysis (IPA; QIAGEN, Redwood City, CA, USA). Significant associations (*p*-value < 0.05) to canonical pathways, gene functions, and up-stream regulators were generated in IPA for each pairwise comparison using significant DE genes (q-value < 0.05, |log_2_FC| ≥ 1.0). Associated pathways and down-stream functions were filtered based on z-score to identify those with predicted activation (z-score ≥ 2) or inhibition (z-score ≤ − 2).

### High throughput qPCR validation

Differential expression from DESeq2 was validated using the Biomark HD system (Fluidigm Corp., San Francisco, CA, USA) to perform high throughput quantitative PCR (qPCR). Primers were selected for 16 genes that had a range of expression patterns, including both up- and down-regulation, and both significant and nonsignificant DE (see Additional file 8 for the complete list of primers). Two stably expressed genes (hexose-6-phosphate dehydrogenase (*H6PD*) and ribosomal protein L4 (*RPL4*)) were also included as references. All primer sets were confirmed previously on the Biomark system [28, 69, 82].

Using the same isolate as the library construction, 50 ng of DNase-treated RNA/sample (*n* = 4 samples/treatment/line) was used to generate cDNA with the Reverse Transcription Master Mix kit (Fluidigm Corp.). Only 12 ng of RNA was available for use as input for cDNA synthesis from three samples (B2009, B2609, and B2619). Target cDNA within each sample was then pre-amplified in 12 cycles of multiplexed PCR using the Pre-amplification Master Mix kit (Fluidigm Corp.). Pre-amplified products were treated with Exonuclease I (New England Biolabs, Ipswich, MA, USA) to remove excess nucleotides (dNTPs and primers) and were diluted 10-fold with TE buffer before use on the Integrated Fluidic Circuit (IFC). Gene expression was assayed using the 192.24 GE Delta Gene Sample and Assay Reagent kit (Fluidigm Corp.) and SsoFast EvaGreen Supermix with Low ROX (Bio-Rad Laboratories, Inc., Hercules, CA, USA). Master-mixes containing each sample and primer pair were generated according to the manufacturer’s protocol and transferred into the inlets of a 192.24 IFC (Fluidigm Corp.); each cDNA sample was run in triplicate. The RX loading station (Fluidigm Corp.) was used to load the samples and primers into the microfluidic circuit and then the Biomark HD was used for thermocycling, which comprised: a hot start at 95 °C for 1 min, 30 cycles of 96 °C for 5 s, 60 °C for 20 s, and a melting curve generated by ramping from 60 °C to 95 °C (1 °C increases; 3 s/step).

### qPCR data analysis

Fluidigm Real-Time PCR Analysis software version 4.3.1 (Fluidigm Corp.) was used to visualize raw qPCR results, calculate the threshold cycles (*C*t) for each reaction, and filter the data according to Fluidigm QC thresholds and the melting curves for each primer set. Samples were excluded from any gene where less than 2 *C*t values were successfully measured for a triplicate or if their *C*t values had a standard deviation greater than 2. Sample B2619 was removed from the dataset entirely due to overly variable results for most genes (standard deviation > 2). Mean *C*t values (of the triplicates) were calculated within each sample for each gene. For the 16 test genes, expression was normalized to delta *C*t (Δ*C*t) using the geometric mean of *H6PD* and *RPL4* and mean Δ*C*t values were determined for each group (line + treatment). Relative expression levels (fold change) were calculated for each comparison between groups using the delta delta *C*t (2^-ΔΔ*C*t^) method [83]. Fold changes were log transformed (log_2_FC) and used to determine pairwise correlations and the R^2^ (linear fit) between RNA-seq and qPCR using JMP Pro 12.0.1 (SAS Institute, Inc., Cary, NC, USA).

## Additional files

Additional file 1: Bird information, experimental design, read QC and mapping for each bursal dataset. (XLSX 23 kb)

Additional file 2: Majority of corrected reads map to the chicken genome. Categorizes the results of read alignment, with the number of reads presented within line/treatment group and the percentage of reads for the total dataset. (TIF 1768 kb)

Additional file 3: Significant differential expression in each comparison between treatments or chicken lines. Contains DESeq2 results for all significant DE genes (q-value < 0.05, |log_2_FC| ≥ 1.0). (XLSX 1400 kb)

Additional file 4: Correlations between log_2_FC from RNA-seq and Biomark qPCR. Compares log_2_FC from RNA-seq and qPCR for contrasts between treatments in broiler and Fayoumi. Panels show all data or each specific treatment comparison and provide the corresponding pairwise correlations and R^2^ of the linear fit. (TIF 5030 kb)

Additional file 5: Significant functional associations for each treatment compared to Thermoneutral + Saline. Lists significant functions (*p*-value < 0.05; |z-score| ≥ 2) and associated significant DE genes for each comparison. (XLSX 29 kb)

Additional file 6: Examples of heat stress and LPS interaction types. Shown using DE from Thermoneutral + LPS (white) and Heat + LPS (gray) in the Fayoumi bursa. A. Suppressed genes: significant in the inter-treatment comparison (Heat + LPS compared to Thermoneutral + LPS) and in Thermoneutral + LPS, but not significant or reversed direction in Heat + LPS. B. Unaffected genes: significant in Heat + LPS and Thermoneutral + LPS, but not in the inter-treatment comparison. C. Induced genes: significant in the inter-treatment comparison and Heat + LPS, but not Thermoneutral + LPS. Differential expression (DE), lipopolysaccharide (LPS). (TIF 1425 kb)

Additional file 7: Significant differential expression between broiler and Fayoumi. Shows overlap in significant DE genes (q-value < 0.05, |log_2_FC| ≥ 1.0) in comparisons of broiler to Fayoumi. (TIF 1817 kb)

Additional file 8: Primers for qPCR validation of differential expression using the Biomark HD system. (DOCX 18 kb)

## Abbreviations

BMDCs: Bone marrow derived dendritic cells
***C*****t**: Threshold cycle
DE: Differential expression
FDR: False Discovery Rate
GALT: Gut associated lymphoid tissue
IFC: Integrated Fluidic Circuit
IPA: Ingenuity Pathway Analysis
ISU: Iowa State University
log_2_FC: log_2_ fold change
LPS: Lipopolysaccharide
PCA: Principal component analysis
qPCR: Quantitative polymerase chain reaction
RIN: RNA Integrity Number
