# Supplementary figures and images for: Immunomodulatory effects of heat stress and lipopolysaccharide on the bursal transcriptome in two distinct chicken lines
Source: BMC Genomics. 2018 Aug 30;19:643. doi: 10.1186/s12864-018-5033-y (PMC6117931; doi:10.1186/s12864-018-5033-y)

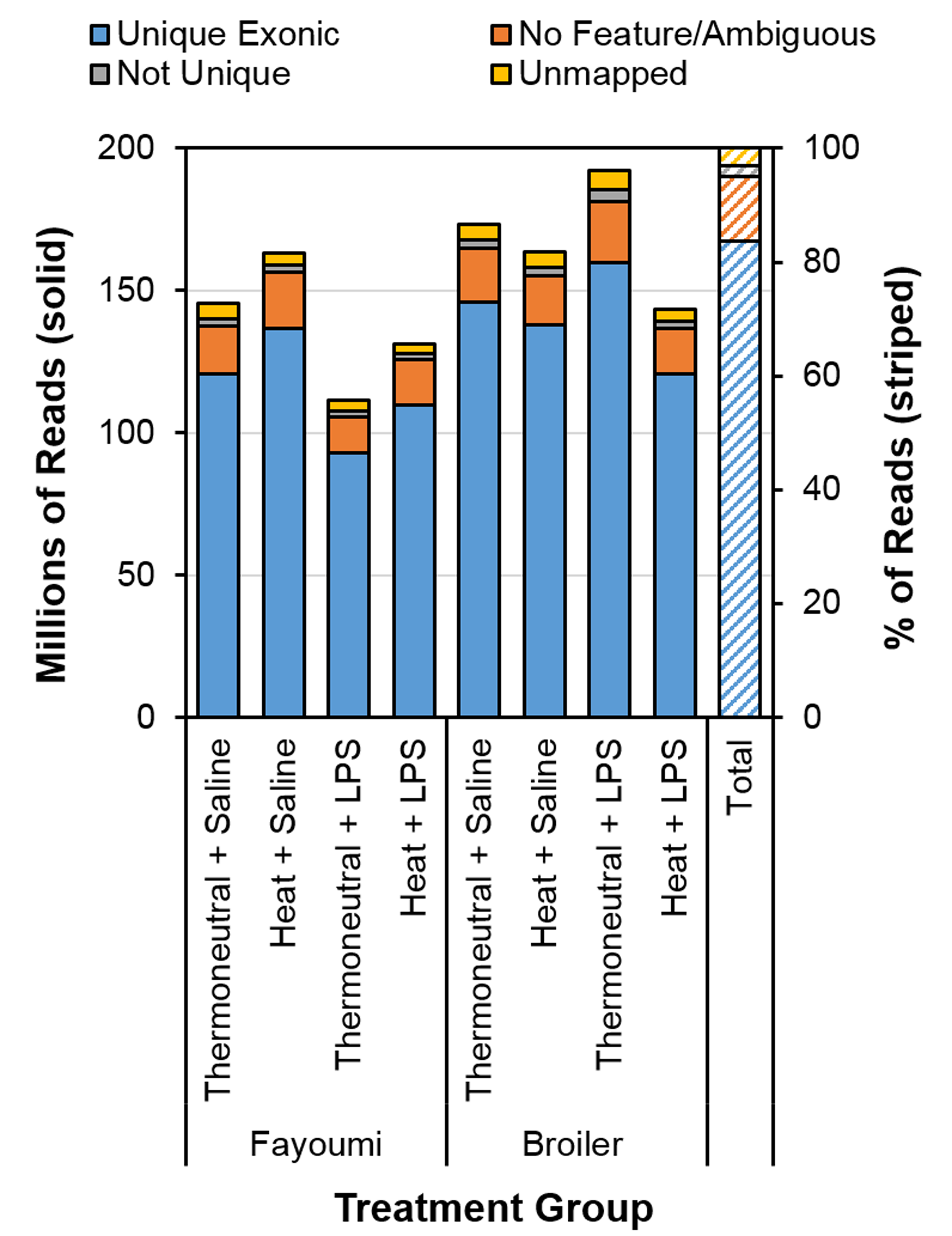

Supplement: Supplementary file 2 — Majority of corrected reads map to the chicken genome. Categorizes the results of read alignment, with the number of reads presented within line/treatment group and the percentage of reads for the total dataset. (TIF 1768 kb) [file 12864_2018_5033_MOESM2_ESM.tif]

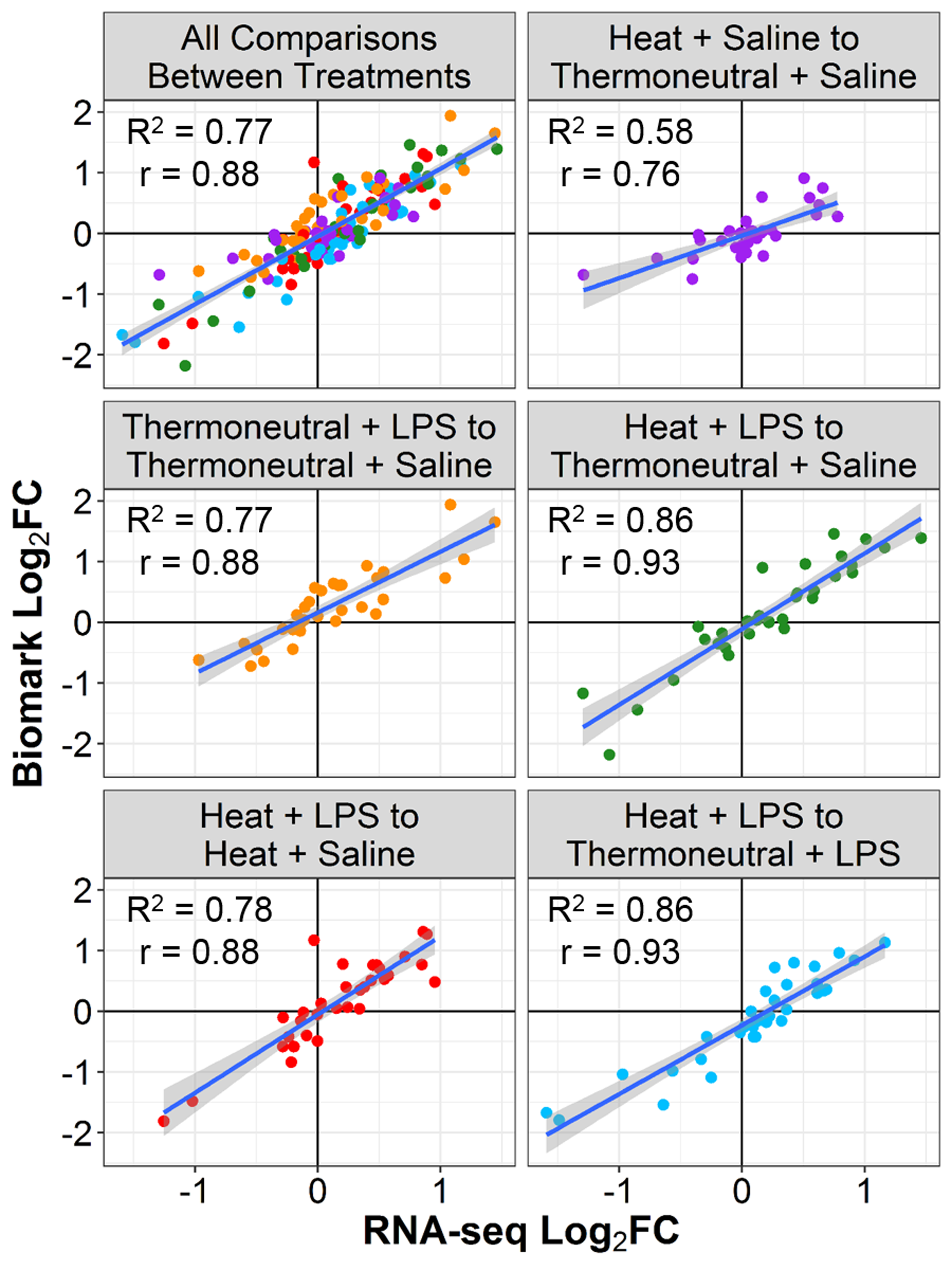

Supplement: Supplementary file 4 — Correlations between log2FC from RNA-seq and Biomark qPCR. Compares log2FC from RNA-seq and qPCR for contrasts between treatments in broiler and Fayoumi. Panels show all data or each specific treatment comparison and provide the corresponding pairwise correlations and R2 of the linear fit. (TIF 5030 kb) [file 12864_2018_5033_MOESM4_ESM.tif]

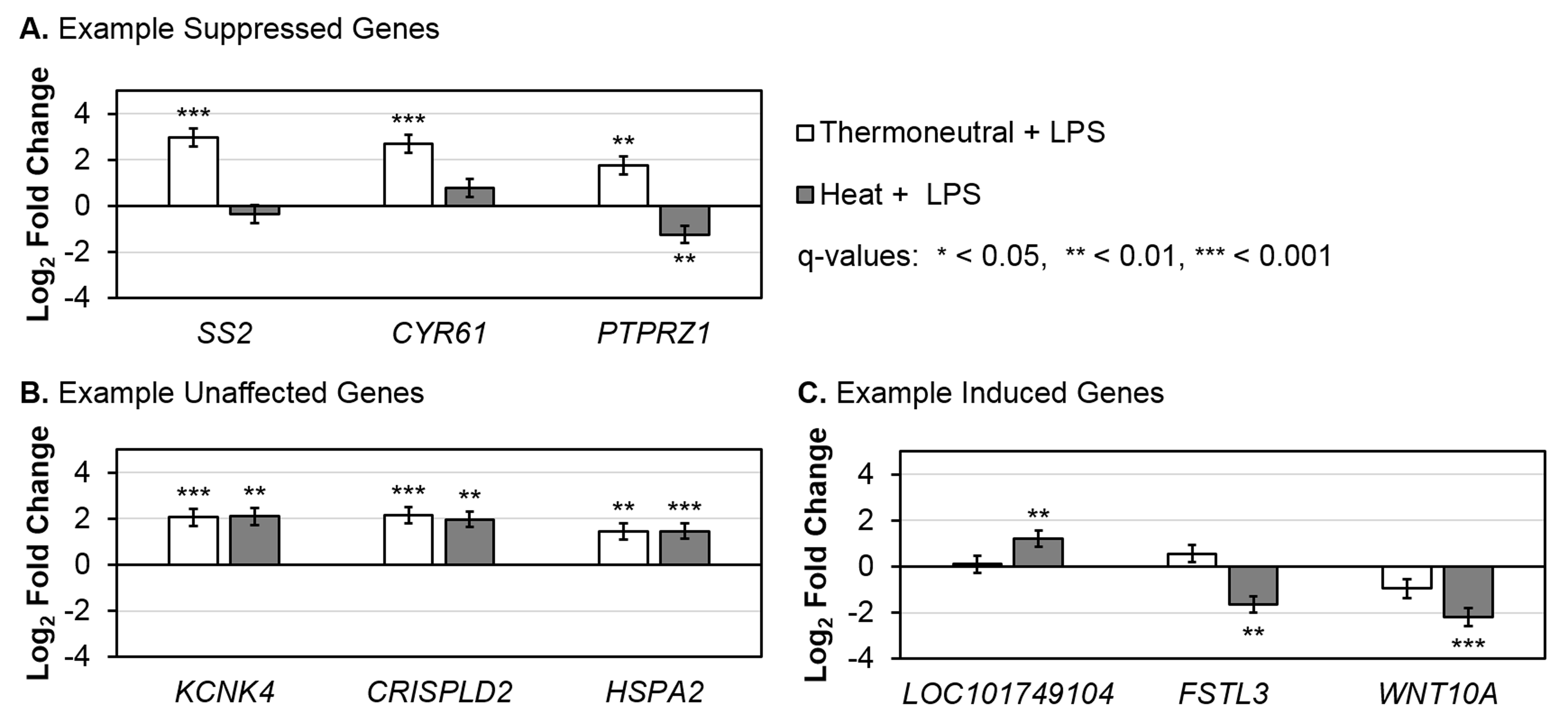

Supplement: Supplementary file 6 — Examples of heat stress and LPS interaction types. Shown using DE from Thermoneutral + LPS (white) and Heat + LPS (gray) in the Fayoumi bursa. A. Suppressed genes: significant in the inter-treatment comparison (Heat + LPS compared to Thermoneutral + LPS) and in Thermoneutral + LPS, but not significant or reversed direction in Heat + LPS. B. Unaffected genes: significant in Heat + LPS and Thermoneutral + LPS, but not in the inter-treatment comparison. C. Induced genes: significant in the inter-treatment comparison and Heat + LPS, but not Thermoneutral + LPS. Differential expression (DE), lipopolysaccharide (LPS). (TIF 1425 kb) [file 12864_2018_5033_MOESM6_ESM.tif]

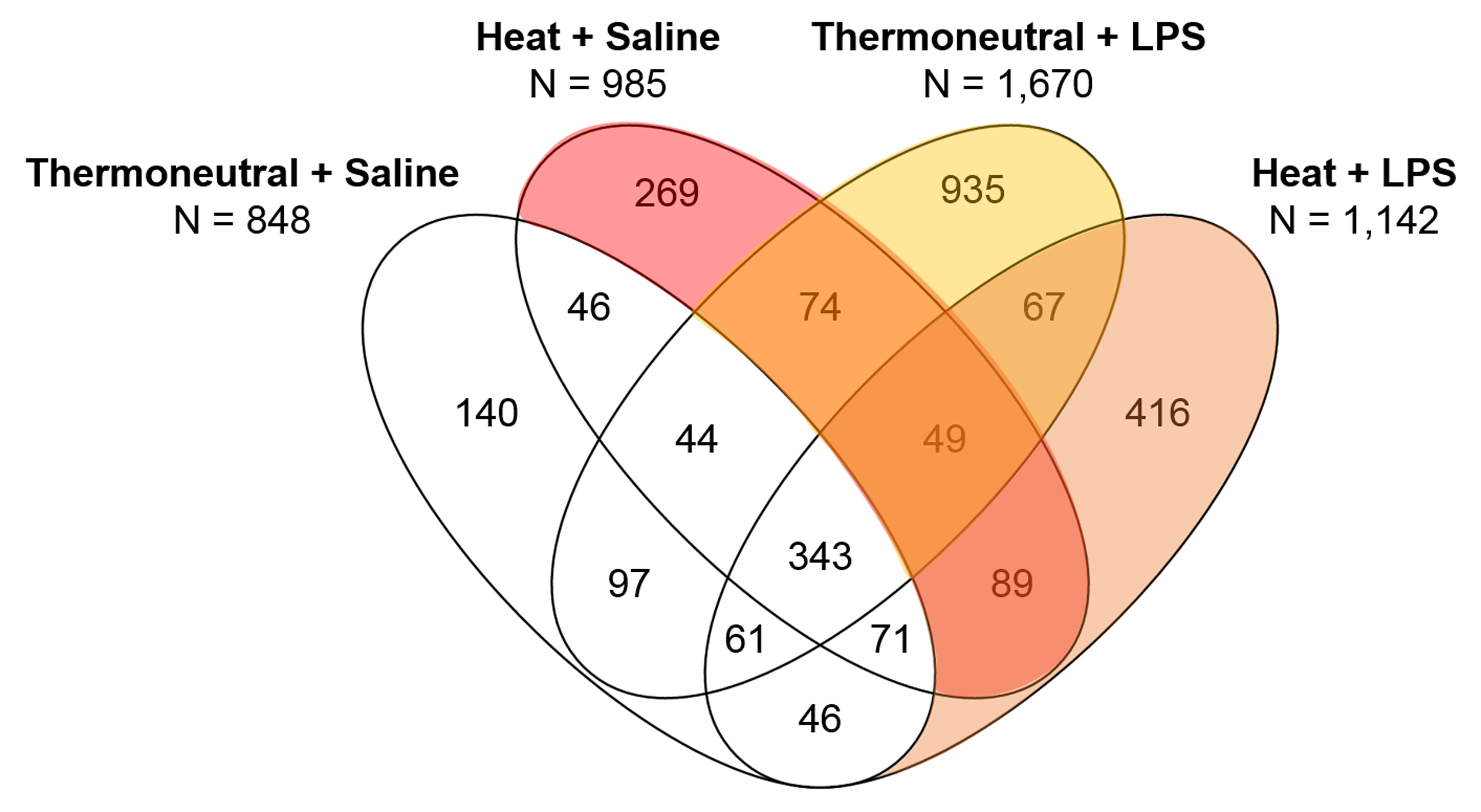

Supplement: Supplementary file 7 — Significant differential expression between broiler and Fayoumi. Shows overlap in significant DE genes (q-value < 0.05, |log2FC| ≥ 1.0) in comparisons of broiler to Fayoumi. (TIF 1817 kb) [file 12864_2018_5033_MOESM7_ESM.tif]
