# Supplementary material for: Immunomodulatory effects of heat stress and lipopolysaccharide on the bursal transcriptome in two distinct chicken lines
Source: BMC Genomics. 2018 Aug 30;19:643. doi: 10.1186/s12864-018-5033-y (PMC6117931; doi:10.1186/s12864-018-5033-y)
Supplement: Supplementary file 8 — Primers for qPCR validation of differential expression using the Biomark HD system. (DOCX 18 kb) [file 12864_2018_5033_MOESM8_ESM.docx]

**Additional file 8.** Primers for qPCR validation of differential expression using the Biomark HD system.

| **Gene** | **Entrez ID** | **Forward (5’-3’)** | **Reverse (5’-3’)** | **Amplicon (bp)** | **Ref.^1^** |
| --- | --- | --- | --- | --- | --- |
| *BAG3* | 423931 | ACCACAACAGCCGAACCA | GATGGGCCATTTGCTGATGAC | 88 | A-B |
| *CASP3* | 395476 | CGCTCAGGGGAAGATGTATCA | CCAGAGTCCACAGACTTGCTA | 86 | A |
| *CASP9* | 426970 | TTTCAGGTCCCTGTGCTTCC | TTCCGCAGCTCCACATCAA | 75 | A-B |
| *CCL4* | 395468 | CCTCATCCAGAGGCACTACA | GCTTGACGCTCTGCAGGTA | 134 | A-C |
| *CD40* | 395385 | AGCCTGGTGATGCTGTGAA | CTCACAGGGTGTGCAGACA | 134 | A-C |
| *H6PD* | 428188 | ATGTACCGGGTGGACCACTA | AACTGACGGTTCTGATCTCGAAA | 77 | A-C |
| *HSP90AA1* | 423463 | ACACATGCCAACCGCATTTA | CCTCCTCAGCAGCAGTATCA | 76 | A |
| *HSPA2* | 423504 | CCACCATTCCCACCAAACAA | ATACACCTGGACGAGGACAC | 77 | A-C |
| *HSPH1* | 418917 | GTAGTTTCGTTCGGCTCCAA | CTGTGTTGTGGGCATGAGTAA | 79 | A-B |
| *IFNG* | 396054 | AACCTTCCTGATGGCGTGAA | GCTTTGCGCTGGATTCTCAA | 86 | B-C |
| *IL10* | 428264 | CCTGCGAGAAGAGGAGCAAA | GGCTTTGTAGATCCCGTTCTCA | 80 | B-C |
| *IL18* | 395312 | CGTGGCAGCTTTTGAAGATGTA | CTGAATGCAACAGGCATCCC | 117 | A, C |
| *IL1B* | 395196 | TGCTTCGTGCTGGAGTCAC | GGCATCTGCCCAGTTCCA | 137 | A-C |
| *IL8L2* | 396495 | CCCCACTGCAAGAATGTTGAAA | GTGCCTTTACGATCAGCTGTAC | 100 | A-B |
| *NOS2* | 395807 | GGACCGAGCTGTTGTAGAGATA | AGCAGCTGAGTGATGATCCA | 88 | A-C |
| *MyD88* | 420420 | GAACGTGTGTGTGGTCCATTA | TGAAATGACGACCACCATCC | 71 | A |
| *RPL4* | 415551 | TTCTGCCTTGGCAGCATCA | AGGAAGTTCTGGGATCTCCTCA | 79 | B-C |
| *TLR4* | 417241 | CCTGCTGGCAGGATGCA | TGTTCTGTCCTGTGCATCTGAA | 77 | B-C |

^1^ References: A [82], B [69], C [28].
